# Acknowledgement to the reviewers of GMS Journal for Medical Education

**DOI:** 10.3205/zma001725

**Published:** 2025-02-17

**Authors:** Martin R. Fischer, Götz Fabry

**Affiliations:** 1 GMS Journal for Medical Education, Chief Editor, Erlangen, Germany; 2 University Hospital, LMU Munich, Institute for Medical Education, Munich, Germany; 3 Albert-Ludwig-Universität Freiburg, Institut für Medizinische Psychologie und Soziologie, Freiburg, Germany

## Acknowledgement

We gratefully acknowledge the valuable contribution of the following reviewers, who supported our Journal in 2024. We are looking forward to continuing our collaboration!

## Reviewers in alphabetical order

- Helmut Ahrens, Münster
- Tobias Albrecht, Tübingen
- Attila Altiner, Heidelberg
- Matthias Angstwurm, München
- Johanna Anneser, München
- Jana Aulenkamp, Essen
- Joy Backhaus, Würzburg
- Rene Ballnus, Stockholm (Sweden)
- Katrin Balzer, Lübeck
- Ivan Bank, Amsterdam (Netherlands)
- Yann-Nicolas Batzler, Düsseldorf
- Daniel Bauer, Bern (Switzerland)
- Martin Baumann, Aachen
- Ronja Behrend, Berlin
- Friederike Bennett, Berlin
- Dora Bernigau, Leipzig
- Annette Bernloehr, Bielefeld
- Henrike Besche, Boston (MA) (USA)
- Silke Biller, Basel (Switzerland)
- Kerstin Bitter, Halle/Saale
- Anja Bittner, Bielefeld
- Mozhgan Bizhang, Witten
- Martin Boeker, München
- Raphael Bonvin, Fribourg (Switzerland)
- Susanne Borgmann, Göttingen
- Claudia Brandt, Frankfurt/Main
- Beate Brem, Bern (Switzerland)
- Johanna Büchel, München
- Andreas Burger, Bochum
- Dogus Darici, Münster
- Iris Demmer, Göttingen
- Carsten Döing, Düsseldorf
- Ivo Dönnhoff, Heidelberg
- Sabine Drossard, Würzburg
- Christine Dunger, Witten
- Nina Eckhoff-Heindl, Köln
- Jan P. Ehlers, Witten
- Bettina Engel, Erlangen
- Malvin Escher, Heidelberg
- Mirjam Faissner, Berlin
- Peter Frey, Bern (Switzerland)
- Sebastian Friedrich, Freiburg i. Brsg.
- Jana Fritsche, München
- Olaf Fritze, Tübingen
- Pia Natalie Gadewoltz, Bielefeld
- Julia Gärtner, Hamburg
- Kirsten Gehlhar, Oldenburg
- Sabine Gehrke-Beck, Berlin
- Susanne Gerhardt-Szép, Frankfurt/Main
- Marianne Giesler, Freiburg i. Brsg.
- Katharina Glassen, Heidelberg
- Michael Goller, Kassel
- Katja Götz, Lübeck
- Heide Götze, Leipzig
- Joachim Graf, Tübingen
- Miriam Grapp, Heidelberg
- Veronika Grosse-Holz, Heidelberg
- Julia Grundnig, Wien (Austria)
- Markus Gulich, Ulm
- Stefan Gysin, Luzern (Switzerland)
- Eckhart G. Hahn, Erlangen
- Wolfgang Hampe, Hamburg
- Laura Hanke, Mainz
- Mike Hänsel, Dresden
- Roman Hari, Bern (Switzerland)
- Rainer Haseneder, München
- Andrea Heinzmann, Freiburg i. Brsg.
- Linn Hempel, Halle/Saale
- Marit Herbolzheimer, Murnau
- Doreen Herinek, Berlin
- Alina Herrmann, Heidelberg
- Monika Himmelbauer, Wien (Austria)
- Barbara Hinding, Mainz
- Johanna Hissbach, Hamburg
- Tanja Hitzblech, Bern (Switzerland)
- Stefan Höfer, Innsbruck (Austria)
- Nicole Hoffmann, Koblenz
- Ylva Holzhausen, Berlin
- Christopher Holzmann-Littig, Friedberg
- Angelika Homberg, Mannheim
- Luke Hopf, Hamburg
- David Hörburger, St. Gallen (Switzerland)
- Claudia Hornberg, Bielefeld
- Johanna Huber, München
- Bert Huenges, Bochum
- Karin Huth, München
- Barbara Jömann, Bochum
- Sylvia Kaap-Fröhlich, Zürich (Switzerland)
- Martina Kadmon, Augsburg
- Julius Josef Kaminski, Berlin
- Yassin Karay, Köln
- Rolf Kienle, Berlin
- Claudia Kiessling, Witten
- Jana Renate Kirchberger, Neuruppin
- Christin Kleinsorgen, Hannover
- Fee Klupp, Heidelberg
- Katharina Knie, Freiburg i. Brsg.
- Harald Knof, Tübingen
- Thomas Kollewe, Frankfurt/Main
- Andrzej Kononowicz, Krakow (Poland)
- Veronika Kopp, München
- Karina Korecky, München
- Mirjam Körner, Bern (Switzerland)
- Marco Kuchenbaur, Augsburg
- Susanne Kühl, Ulm
- Thomas Kühlein, Erlangen
- Sandy Kujumdshiev, Leipzig
- Raphael Kunisch, Augsburg
- Silke Kuske, Düsseldorf
- Nathalie Laissue, Basel (Switzerland)
- Bea Langhoff, Heidelberg
- Christoph Lanzen, Mainz
- Jan Lauter, Heidelberg
- Bettina Leeuw, Bielefeld
- Ronny Lehmann, Heidelberg
- Sabine Ludwig, Innsbruck (Austria)
- Benedikt Luka, Hannover
- Björn Lütcke, Erlangen
- Gabriele Lutz, Witten
- Ruth Mächler, München
- Frederik Mader, Nittendorf
- Julia Mahal, Heidelberg
- Cornelia Mahler, Tübingen
- Daniela Mäker, Essen
- Hartwig Marung, Hamburg
- Jan Matthes, Köln
- Benjamin Mayer, Ulm
- Ana Mazur, Bielefeld
- Parisa Moll-Khosrawi, Hamburg
- Sören Moritz, Köln
- Ashley Mullen, Houston (TX) (USA)
- Christiane Müller, Göttingen
- Martin Müller, Bern (Switzerland)
- Beate Müller, Köln
- Andrea Neher, Bern (Switzerland)
- Felix Nickel, Hamburg
- Christoph Nikendei, Heidelberg
- Lukas Nock, Mannheim
- Dino Carl Novak, Berlin
- Heidi Oberhauser, Innsbruck (Austria)
- Udo Obertacke, Mannheim
- Dorothea Penders, Berlin
- Harm Peters, Berlin
- Swetlana Philipp, Jena
- Sarah Prediger, Hamburg
- Wolfgang Prodinger, Innsbruck (Switzerland)
- Christina Quandt, Hannover
- Alexander Rahman, Hannover
- Florian Recker, Bonn
- Maike Reingen, Dresden
- Stefan Reinsch, Neuruppin
- Annette Rogge, Helgoland
- Carolin Rolle, Augsburg
- Ivo Rollmann, Heidelberg
- Bernd Romeike, Rostock
- Sabine Salloch, Hannover
- Thorsten Schäfer, Bochum
- Rainhild Schäfers, Münster
- Elisabeth Schaper, Hannover
- Stefan Schauber, Oslo (Norway)
- Christian Scheffer, Witten
- Theresa Scherer, Bern (Switzerland)
- Kristina Schick, Dresden
- Claudia Schlegel, Bern (Switzerland)
- Anita Schmidt, Erlangen
- Achim Schneider, Ulm
- Eva Schönefeld, Münster
- Juliane Schopf, Münster
- Teresa Schreckenbach, Frankfurt/Main
- Sylvia Schrempf, Tübingen
- Jeannine Schübel, Dresden
- Sven Schulz, Jena
- Christina Schut, Gießen
- Katrin Schüttpelz-Brauns, Mannheim
- Mathias Schütz, München
- Simon Schwill, Heidelberg
- Rebecca Schwoch, Hamburg
- Alexander Peter Schwoerer, Hamburg
- Monika Sennekamp, Frankfurt/Main
- Thomas Shiozawa-Bayer, Tübingen
- Jan Siebenbrock, Münster
- Anne Simmenroth, Würzburg
- Melanie Simon, Aachen
- Dirk Sommerfeldt, Hamburg
- Cord Spreckelsen, Jena
- Babette Stadler-Werner, Regensburg
- Sandra Steffens, Hannover
- Beate Stock-Schröer, Witten
- Jessica Stokes-Parish, Gold Coast (Australia)
- Christoph Stosch, Köln
- Michaela Stratmann, Witten
- Ulrike Subotic, Basel (Switzerland)
- Paul Supper, Wien (Austria)
- Christian Thrien, Köln
- Judith Ullmann-Moskovits, Frankfurt/Main
- Mirdita Useini, Zürich (Switzerland)
- Ursula Walkenhorst, Osnabrück
- Gesine Wegner, Dresden
- Birgitta Weltermann, Bonn
- Birgit Wershofen, München
- Bärbel Wesselborg, Düsseldorf
- Swantje Wienand, Bremen
- Tugba Zahn, Frankfurt/Main
- Stephan Ziegenhorn, Bern (Switzerland)
- Isabel Zorn, Köln

## Authors’ ORCIDs

- Martin R. Fischer: [0000-0002-5299-5025]
- Götz Fabry: [0000-0002-5393-606X]

